# Trial of Remote Continuous versus Intermittent NEWS monitoring after major surgery (TRaCINg): protocol for a feasibility randomised controlled trial

**DOI:** 10.1186/s40814-018-0299-z

**Published:** 2018-06-11

**Authors:** C. L. Downey, J. Croft, H. Buckley, R. Randell, J. M. Brown, D. G. Jayne

**Affiliations:** 1 Leeds Institute of Biomedical and Clinical Sciences, Clinical Sciences Building, St. James’s University Hospital, University of Leeds, Level 7 Clinical Sciences Building, Leeds, LS9 7TF UK; 2 Clinical Trials Research Unit, Leeds Institute of Clinical Trials Research, University of Leeds, Leeds, LS2 9NL UK; 3 School of Healthcare, Baines Wing, University of Leeds, Leeds, LS2 9JT UK

**Keywords:** Continuous, Early warning score, Vital signs, Monitoring, Surgery, Complications

## Abstract

**Background:**

Despite medical advances, major surgery remains high risk. Up to 44% of patients experience postoperative complications, which can have huge impacts for patients and the healthcare system. Early recognition of postoperative complications is crucial in reducing morbidity and preventing long-term disability. The current standard of care is intermittent manual vital signs monitoring, but new wearable remote monitors offer the benefits of continuous vital signs monitoring without limiting the patient’s mobility. The aim of this study is to evaluate the feasibility, acceptability and clinical impacts of continuous remote monitoring after major surgery.

**Methods:**

The study is a randomised, controlled, unblinded, parallel group, feasibility trial. Adult patients undergoing elective major surgery will be invited to participate if they have the capacity to provided informed, written consent and do not have a cardiac pacemaker or an allergy to adhesives. Participants will be randomly assigned to receive continuous remote monitoring and normal National Early Warning Score (NEWS) monitoring (intervention group) or normal NEWS monitoring alone (control group). Continuous remote monitoring will be achieved using the SensiumVitals® wireless patch which is worn on the patient’s chest and monitors heart rate, respiratory rate and temperature continuously and alerts the nurse when there is deviation from pre-set physiological norms. Participants will be followed up throughout their hospital admission and for 30 days after discharge. Feasibility will be assessed by evaluating recruitment rate, adherence to protocol and randomisation, and the amount of missing data. The acceptability of the patch to nursing staff and patients will be assessed using questionnaires and interviews. Clinical outcomes will include time to antibiotics in cases of sepsis, length of hospital stay, number of critical care admissions and rate of readmission within 30 days of discharge.

**Discussion:**

Early detection and treatment of complications minimises the need for critical care, improves patient outcomes, and produces significant cost savings for the healthcare system. Remote continuous monitoring systems have the potential to allow earlier detection of complications, but evidence from the literature is mixed. Demonstrating significant benefit over intermittent monitoring to offset the practical and economic implications of continuous monitoring requires well-controlled studies in high-risk populations to demonstrate significant differences in clinical outcomes; this feasibility trial seeks to provide evidence of how best to conduct such a confirmatory trial.

**Trial registration:**

This study is listed on the ISRCTN registry with study ID ISRCTN16601772.

## Background

Patients having major surgery are at high risk of complications, some of which can be life-threatening. Rates of complications have been found to be as high as 33–44% in patients undergoing surgery for gastrointestinal cancers [1]. Patients who develop postoperative complications become progressively unwell, often over a short period of time. Early recognition of postoperative complications is crucial in reducing morbidity and preventing long term-disability; for patients with septic shock, there is an 8% increase in mortality for every hour of delay in antibiotic administration [2].

The more unwell a patient becomes, the more likely they are to require higher level care, on either high-dependency units (HDU; level II) or intensive care units (ICU; level III). Escalation of care comes at significant cost to both the patient and the health service and is associated with worse patient outcomes. The average cost of a level I (general ward) bed is £433/day, as compared to £1033/day for a HDU bed, and £1351/day for an intensive care bed [3]. Early detection and treatment of complications minimises the need for level II/III care, improves patient outcomes and produces significant cost savings.

One of the ways patients are monitored for complications is by recording on a chart their vital signs. The vital signs are used to form a score, the National Early Warning Score (NEWS), which can alert if the patient becomes unwell. The vital signs included in the NEWS score are blood pressure, heart rate, breathing rate, oxygen saturation, level of consciousness and temperature [4]. The NEWS score also takes into account the need for supplementary oxygen to maintain a patient’s saturations. Typically, in the postoperative period, NEWS will be calculated half hourly for the first few hours, and if the patient remains stable, the frequency will decrease to 2-hourly and then 4-hourly, until the patient is ready for discharge when the NEWS may be recorded only twice a day.

Although NEWS has proven benefit, it suffers from several drawbacks. A 2012 study evaluated early warning scores in patients 48 h before an adverse event [5]. Of patients, 81% had a score indicative of deterioration, but recordings were ‘mostly incomplete’ with respiratory rate documented in ‘only 30% to 66%’. NEWS relies on manual observations, is time-consuming, and open to user interpretation. Vital signs are taken at predetermined intervals (typically 4-hourly), with patient deterioration possible between recordings. It has been suggested that the gap between observations is one of the primary failings of the NEWS system [6].

A solution to the problem of inadequate monitoring frequency is continuous monitoring at the bedside. Continuous monitoring is used in level II/III care, but is limited by “hard-wired” equipment, which tethers the patient to the bed space. This hinders patient mobility and potentially slows their recovery. One study tested ICU-style monitoring on a general ward and found that only 16% of patients remained connected in a 72-h period [7].

A number of systems have been designed that combine the benefits of a wearable, wireless patch with continuous monitoring of vital signs. One such device is the SensiumVitals® which monitors heart rate, respiratory rate and temperature continuously. The data are transmitted wirelessly every 2 min to a mobile device carried by the nurse which alerts when there is deviation from pre-set physiological norms.

It is hypothesised that the remote continuous monitoring system, as an adjunct to standard NEWS monitoring, will allow earlier detection of postoperative complications. This should reduce morbidity, which in turn should result in a decreased need for high dependency/intensive care.

Studies evaluating continuous monitoring of multiple vital signs parameters have shown mixed results. There is a preponderance of observational studies, which means that causal associations between interventions and patient outcomes have to be interpreted with care. An industry-funded controlled before-and-after study of 7643 patients [8] found that continuous monitoring on a medical-surgical unit was associated with a decrease in total ICU days, but the rate of ICU admission was unchanged.

The three largest randomised controlled trials in this area show conflicting results but share common limitations. A randomised controlled trial of 402 high-risk medical and surgical patients found that continuous multi-parameter monitoring showed no effect on adverse events or mortality [9]. However, only 16% of the patients were continuously monitored for the full 72 h intended. Patient and staff compliance may have influenced the impact of the interventions. This trial was also underpowered to detect differences in clinical outcome measures given the complex nature of the intervention under investigation.

Demonstrating significant benefit over intermittent monitoring to offset the practical and economic implications of continuous monitoring is difficult and requires large, well-controlled studies in high-risk populations to demonstrate significant differences in clinical outcomes, such as critical care admissions. Given the complexity of the intervention, before a definitive trial is designed, there is the need for a feasibility study focussed not only on clinical outcome measures but also patient and nursing acceptability and compliance. Compliance is unpredictable yet crucial to the adequate assessment of the intervention. If the patient or nursing staff refuse to engage with the monitoring device, this will negate the need for a definitive trial. In addition, a feasibility study will allow the identification of barriers to recruitment and protocol adherence and allow optimisation of the trial design and the technology itself.

### Aims

The main aim is to determine the feasibility of performing a large-scale randomised controlled trial of continuous remote monitoring after major surgery. A secondary objective is to evaluate the safety, potential efficacy and acceptability of a wearable, remote monitoring system for patients after major surgery, as compared to standard monitoring with the NEWS system alone.

### Trial design

This is a single-centre, feasibility, randomised, controlled, parallel group trial of continuous remote vital signs monitoring for patients who have undergone major elective general surgery. Participants will be individually randomised on a 1:1 basis to receive either remote monitoring plus NEWS or monitoring by NEWS alone. Randomisation will use random permuted blocks and be stratified by ASA (American Society of Anaesthesiologists comorbidity score) and gender.

## Methods

### Study setting

All participants will be recruited from St James’s University Hospital, Leeds, UK.

### Inclusion criteria

- Patients who are undergoing elective surgery
- Patients who have the capacity to provide informed, written consent on admission
- All ages ≥ 18 years

### Exclusion criteria

- Patients who have undergone emergency surgery
- Allergy to adhesives on electrodes
- Cardiac pacemaker in situ

### Recruitment

Patients will be selected on the basis that they are undergoing elective major abdominal surgery and are anticipated to return to one of the participating wards afterwards. Types of surgery can include, but are not limited to, colorectal, upper gastrointestinal, liver, pancreas and biliary surgeries.

Table 1 illustrates the participant timeline for this study. Patients will be identified, recruited and consented for inclusion in the trial on the day of their surgery. This will take place on the General Surgical Admissions Lounge where patients attend on the morning of their elective procedures.

**Table 1:**
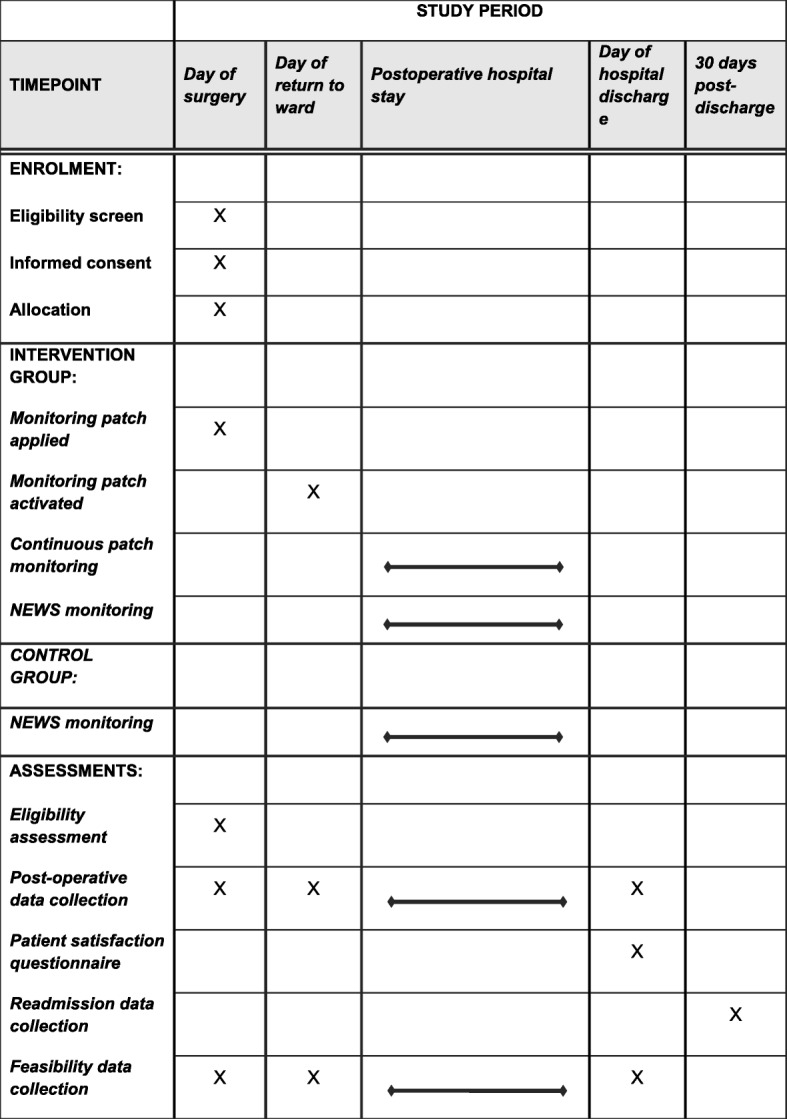
Schedule of enrolment, interventions, and assessments

The patients will be given information in the form of a patient information sheet regarding the study. Following a period of consideration, if they consent to participate in the study, they will be randomised into one of two monitoring arms.

### Randomisation

Following confirmation of eligibility and written informed consent, participants will be randomised into the trial by an authorised member of staff at the research site. Randomisation will be performed centrally using the University of Leeds Clinical Trials Research Unit (CTRU) 24-h randomisation service, either via the telephone or the CTRU website.

Participants will be randomised on a 1:1 basis and will be allocated a unique trial number. Randomisation will be conducted using stratified block randomisation with variable block size with sex (male/female) and ASA grade (grades 1–4) as stratification factors.

The randomisation sequence will be provided by a statistician in the Leeds Clinical Trials Research Unit and computer generated using SAS 9.4 (SAS Institute Inc., USA, 2013). This sequence will be implemented and delivered by programmers through the Leeds CTRU Gen24 system, a dedicated telephone and web-based randomisation service.

### Interventions

Patients randomised to the ‘intervention’ arm will receive a patch and standard NEWS monitoring.

When the patient comes out of theatre, they are nursed in Recovery for a short time before being admitted on to the receiving ward. The patients allocated to receive the remote monitoring will, where possible, have the patch applied in Recovery by a member of the research team. If for any reason the patch cannot be applied in Recovery, it will be applied as soon as possible upon the patient’s return to one of the participating wards. If a participant is admitted to level II/III care after surgery and before returning to a participating ward, they will have the patch applied in Recovery but the patch will only be activated once the patient returns to the participating ward. This will usually be 1–2 days later.

Two colorectal surgery wards will be participating in the study: male and female. The male ward houses 25 beds, whilst the female ward houses 28 beds.

The patch will activate on arrival on the ward, and the patient’s nurse will carry a mobile device to alert them if the vital signs stray outside of normal parameters. Remote monitoring data will also be accessible on the ward computer screens for wider access. There will be no dedicated telemetry screen for the patch data.

Nursing staff will be provided with thorough training before the commencement of the study. This will involve training in the application and removal of the patch, the use of the mobile application and how to acknowledge alerts. If the mobile devices alert the nursing staff to abnormal vital signs, the ensuing clinical response is not mandated, but left to the nurse’s discretion.

In order to monitor alarm burden, the Clinical Fellow assigned to the trial will make daily ward visits and ask the nursing staff about false alerts. The Fellow will then adjust the monitor’s delays and thresholds on an individual basis and according to clinical need, for instance, adjusting the heart rate alert threshold in patients with pre-existing cardiac arrhythmias.

During these daily ward visits, the Fellow and the research nurse will also be responsible for changing expired patches and removing patches upon patient discharge. In order to optimise patient comfort and compliance with the device, it will also be possible to adjust or replace electrodes, including after patient washing.

Patients in the ‘control’ arm will receive standard NEWS monitoring alone. All usual nursing and medical care are permitted within both arms of the trial.

### Blinding

Blinding is not applicable for this study. Neither the patients nor the nursing staff can be blinded to the intervention received. The data collection will be performed by a research nurse and clinical fellow, who will both be administering the monitoring device, and so are necessarily unblinded. However, the objective methods of collecting the outcome data minimise the risk of bias. These data will be taken from the clinical records made by the patients’ usual care teams, including a succession of junior medical staff on rotation, who will be unaware of the study. In addition, the predefined criteria for the outcome measures provide minimal scope for interpretation of their presence or absence by the data collection team. The clinical fellow will be performing the analysis alongside an unblinded statistician.

### Data collection

The patients will remain in their allocated study arm for the duration of their hospital stay. If a remotely monitored patient is moved to a critical care bed during their admission, the remote monitoring will be temporarily suspended pending reinstatement depending whether they return to a participating ward. Every effort will be made to ensure that participants remain in the study arm to which they were originally allocated, and any non-compliance will be recorded.

Patients’ participation in the trial will end when they are discharged from hospital. At this point, remotely monitored patients will be invited to complete a questionnaire regarding their experiences of wearing the patch (see Appendix 1). Information regarding the admission will be collected once the patient has left hospital. Information will also be collected regarding the number of patients who agree to take part in the study, those who do not and the reason for not taking part.

The nursing staff (registered nurses and healthcare assistants) will be invited to complete a paper questionnaire about system usability (see Appendix 2). They will also be invited to undertake a semi-structured interview regarding their experience of providing the new monitoring system based on a purposive sampling strategy, to get the most comprehensive impression of nursing perceptions. This purposive sample will include nurses at various grades and with different experiences of the device.

### Primary outcome measures

- Recruitment rate, including proportion of ineligible patients and reasons for non-consent
- Information on the ideal method of randomisation, which will include calculation of intra-cluster correlation co-efficient to investigate whether there is any inherent clustering in outcomes based on which ward bay a participant is admitted to
- Adherence to protocol, and reasons for non-adherence, as defined by the number of patients who do not receive the correct type of monitoring as per randomisation (and reasons for this) and the number of patients who do not wear the patch for their entire hospital stay or at least 5 days during their admission
- Amount of missing clinical data, as collected on the Case Report Forms, and loss-to-follow-up
- Optimal outcome measures to test effectiveness (see secondary outcome measures)
- Estimation of parameters to input into the sample size calculation for a definitive RCT.

### Secondary outcome measures

- Time to antibiotics in cases of sepsis
- Number of HDU/ICU admissions
- Length of stay in HDU/ICU
- Total length of stay in hospital
- Number of postoperative complications, defined and scored according to the Clavien-Dindo classification of surgical complications [10]
- Number of reinterventions, defined and scored according to the Clavien-Dindo classification of surgical complications [10]
- Patient acceptability using questionnaire scores
- Number of patients not wearing patch for at least 5 days
- Nursing acceptability using questionnaire scores and thematic analysis of interviews
- 30-day readmission rate

### Sample size and expected accrual

As the trial is designed to assess the feasibility of conducting a future definitive large-scale trial, a formal power calculation is not considered appropriate as effectiveness is not being formally evaluated.

According to the findings of Teare et al. [11], at least 120 subjects (60 in each group) will be required in the feasibility RCT to estimate event rates in the intervention arm with adequate precision. Anticipating an estimated consent rate of 30–50%, between 240 and 400 patients will be approached in order to recruit 120 participants.

In addition, it is possible that not all participants will be assigned a bed in a participating ward. Allowing for 20% of patients going to non-participating wards, with the expectation that this is likely to be balanced between the study groups, this necessitates that 300 to 500 patients will need to be approached and approximately 150 participants randomised in order to have monitoring data on 120 participants in total. With a 12-month recruitment period, this equates to 6–10 patients being approached on average per week.

Patients who are not admitted to a participating ward will be classed as ‘drop-out’ due to design and will not be included in the modified intention-to-treat analysis.

### Planned analyses

Analyses have been pre-specified in a statistical analysis plan. The analysis of the primary and secondary outcome measures will take place when all participants have been followed up (i.e. 30 days after the last recruited participant’s date of discharge). No interim analyses are planned but safety data will be presented to the Multidisciplinary Advisory Group at regular intervals.

Analysis will be carried out following the principles of modified intention-to-treat (ITT). The modified ITT (mITT) population will include all participants randomised to the trial, analysed according to the treatment group to which they were randomised, regardless of adherence to the protocol. The mITT population will not include any participants who are classed as ‘drop-out’ due to design (i.e. those who were never admitted to a participating ward).

As this is a feasibility study, no formal comparison between the study arms will be undertaken. Summaries will be produced by subgroup to determine any differences between low- and high-risk patients. High-risk patients will be defined as ASA > 2 undergoing major + surgery or ASA = > 2 with a perioperative critical care admission.

Baseline characteristics will be summarised descriptively overall and by trial arm. No statistical comparison between trial arms will be made.

Quantitative secondary outcome measures will be summarised descriptively using appropriate summary statistics both overall and by trial arm (mean, standard deviation, range and median for continuous outcomes and frequency and percentages for categorical measures). Proportions of missing data will also be presented. Data from patients discharged before the 5 days has elapsed will be censored. Qualitative secondary outcomes including patient acceptability and nurse acceptability will be analysed using a thematic analysis approach.

### Safety

As this is a feasibility study, data will be monitored prospectively by the research team. Any clinical concerns or complications occurring in excess of those normally experienced after this type of surgery will be reported by the Chief Investigator to the Sponsor and appropriate action taken to suspend or terminate the study until such time that patient safety can be assured in line with national guidelines for patient outcomes in surgery.

### Data management

Personal data collection during the study will be handled in accordance with Data Protection Act 1998. All information collected during the course of the trial will be kept strictly confidential.

Information will be held securely on paper and electronically at Leeds Teaching Hospitals NHS Trust and the University of Leeds Clinical Trials Research Unit (CTRU). The study site will maintain a file of essential trial documentation and will keep copies of completed case report forms (CRFs) for the trial. Completed CRFs will be sent to the CTRU for entry onto the secure trial database.

All vital signs data collected by the SensiumVitals® system are stored and retained on the hospital network. The SensiumVitals® system inherits all the hospital security procedures and data backup policies, to ensure data access and servers are secured.

In line with the principles of Good Clinical Practice guidelines, at the end of the trial, data will be securely archived for a minimum of 5 years.

### Data monitoring and validation

Day to day monitoring for completeness and quality of trial data will be conducted centrally at the CTRU by the Data Manager or their delegate. Every effort will be made to ensure that as much data as possible are available and that reasons for unobtainable data are recorded.

For a feasibility study of this nature and duration, a separate Data Monitoring and Ethics Committee is not required. The Multidisciplinary Advisory Group will meet approximately monthly and review screening, recruitment, site monitoring, data quality, protocol compliance and withdrawals.

The data manager (or their delegate) will perform verification of the forms in real time, as data are received, in accordance with the guidelines developed for the study. This will ensure that data are complete, consistent and up-to-date. Key data items will be 100% checked by the data manager or their delegate. In addition, statistical checks will be used to validate the data and check for any missing or inconsistent data.

### Progression criteria

The criteria for progression to a definitive randomised controlled trial will be as follows:

- The recruitment of 120 patients within 12 months who receive monitoring on the trial
- Missing data limited to no more than 20% attrition

### Dissemination

The trial results will be disseminated in a published manuscript and through presentation to clinical and patient and public forums with the aim of reaching healthcare workers and other stakeholders throughout the healthcare system.

## Discussion

Although continuous monitoring is routinely used in high-dependency care, it has practical implications which have to be offset in the general ward setting. Many of the existing clinical studies were limited by the complex nature of the intervention under investigation. Patient and staff compliance may have influenced the impact of the interventions. In order to ensure maximum benefit from continuous monitoring technologies, it is crucial to engage patients and nursing staff in the implementation and, ideally, development of the intervention.

Previous studies have found the main barriers to the implementation of remote monitoring are nursing engagement and alarm burden [8]. In this trial, these issues will be addressed through a concurrent process evaluation. Nurses will be provided with thorough training, and their engagement in the use of the device and perceptions of the adequacy of the training will be explored through the questionnaire and interviews. False alert rates will be monitored on a daily basis and delays and thresholds will be adjusted accordingly. Patient satisfaction will also be assessed in order to optimise the patient’s comfort and compliance with the device.

Demonstrating significant benefit over intermittent monitoring to offset the practical and economic implications of continuous monitoring requires the optimisation of the intervention to the mutual satisfaction of nursing staff and patients alike. This feasibility study will focus on not only clinical outcome measures but also patient and nursing acceptability and compliance with the intervention. This will allow the identification of barriers to recruitment and protocol adherence and allow optimisation of the definitive trial protocol.

### Trial status

At the time of submission, TRaCINg is open to recruitment.

## Appendix 1

**Table 2:** Patient experience questionnaire

Statement	Strongly agree	Agree	Neutral	Disagree	Strongly disagree
Comfort					
The SensiumVitals® Patch was comfortable to wear.					
Quality of Care					
I felt safer because my vital signs were being monitored constantly.					
We welcome any additional comments you may have:					

## Appendix 2

**Table 3:** Modified system usability score

	Strongly disagree				Strongly agree
I think that I would like to use this product again	1	2	3	4	5
I found the product unnecessarily complex	1	2	3	4	5
I thought the product was easy to use	1	2	3	4	5
I think that I would need the support of a technical person to be able to use this product	1	2	3	4	5
I found that the various functions in this product were well integrated	1	2	3	4	5
I thought that there was too much inconsistency in this product	1	2	3	4	5
I would imagine that most people would learn to use this product very quickly	1	2	3	4	5
I found the product very awkward to use	1	2	3	4	5
I felt very confident using the product	1	2	3	4	5
I needed to learn a lot of things before I could get going with this product	1	2	3	4	5

## Abbreviations

ASA: American society of anaesthesiologists (score for pre-operative functional status)
CTRU: Clinical Trials Research Unit
HDU: High-dependency unit
ICU: Intensive care unit
ITT: Intention-to-treat
mITT: Modified intention-to-treat
NEWS: National Early Warning Score
RCT: Randomised controlled trial
